# Genotype-phenotype correlations in *EPCAM*-associated congenital tufting enteropathy: a case report and systematic review

**DOI:** 10.3389/fped.2026.1830464

**Published:** 2026-05-21

**Authors:** Liqian Zhao, Yucan Zheng, Hao Liu, Zhifeng Liu, Yan Lu, Kunlong Yan

**Affiliations:** 1 Department of Gastroenterology, Children’s Hospital of Nanjing Medical University, Nanjing, China; 2 Department of Infection, Children’s Hospital of Nanjing Medical University, Nanjing, China

**Keywords:** congenital tufting enteropathy, EPCAM, genotype-phenotype analysis, intestinal failure, parenteral nutrition

## Abstract

**Background:**

Congenital tufting enteropathy (CTE) is a rare autosomal-recessive enteropathy caused by *EPCAM* variants. While the clinical spectrum ranges from intractable diarrhea to manageable outcomes, the genotype-phenotype correlation remains undefined.

**Methods:**

We report a case of an infant diagnosed with CTE carrying a novel frameshift variant (c.315delT), and present a systematic review of previously published molecularly confirmed cases. Genotypes were stratified into truncating (nonsense, frameshift, large deletions) and non-truncating (missense, splicing, in-frame indel) groups to assess their impact on intestinal failure severity.

**Results:**

The index patient, carrying a homozygous truncating variant, presented with severe malnutrition and intestinal failure. In the pooled cohort (*n* = 150), truncating variants were significantly associated with dependency on total parenteral nutrition (TPN) compared to non-truncating variants (OR 2.37, 95% CI 1.15–4.90, *p* = 0.020). Sensitivity analysis supported this association (OR 2.42, 95% CI 1.07–5.47, *p* = 0.045). Geographically, truncating variants were enriched in the Eastern Mediterranean Region (OR 9.32, *p* < 0.001).

**Conclusions:**

Truncating *EPCAM* variants showed a preliminary but significant clinical association with severe intestinal failure requiring TPN. These findings support early risk stratification for aggressive nutritional management but warrant further validation in prospective cohorts.

## 1 Introduction

Congenital tufting enteropathy (CTE) is an autosomal recessive disorder caused by pathogenic variants in *EPCAM*, typically presenting with intractable diarrhea, malnutrition, and growth failure in early infancy (1). The estimated incidence is 1 in 50,000–100,000 live births, with a higher prevalence in regions with elevated consanguinity (2). *EPCAM* (2p21) encodes a transmembrane glycoprotein essential for epithelial adhesion and barrier homeostasis. Pathogenic variants disrupt intercellular adhesion, leading to hallmarks such as villous atrophy and characteristic epithelial tufts on histology (3, 4).

Clinically, persistent watery diarrhea and failure to thrive are predominant, with a subset of patients presenting with associated congenital anomalies (5). Histopathology findings often include blunted villi, disorganized surface epithelium, and focal epithelial tufts (6). Delayed or missed diagnosis increases the risks of dehydration, malnutrition, and infections, while early recognition and initiation of total parenteral nutrition (TPN) can improve outcomes, albeit with vigilance for TPN-related complications such as liver dysfunction and sepsis (2, 7).

Although CTE is monogenic, its clinical severity spans a broad continuum, ranging from intermittent watery stools that can be managed with enteral nutrition formulas (8) to rapidly progressing intestinal failure or even death (2, 7, 9). Therefore, the genotype-phenotype relationship of *EPCAM* variants and their outcomes remains poorly defined. The breadth of trajectories underscores the need for early risk stratification.

We encountered an infant diagnosed with CTE carrying an early truncating *EPCAM* variant (c.315delT), who experienced rapid clinical deterioration and early death despite supportive care. This clinical observation prompted us to examine whether *EPCAM* variant class is associated with nutritional dependency and clinical outcomes in previously reported molecularly confirmed cases.

## 2 Methods

### 2.1 Information sources and search strategy

This study integrates a novel case of an infant diagnosed with CTE with a PRISMA-guided systematic review and pooled analysis of the literature. A comprehensive search was performed in PubMed and Web of Science from inception to May 1, 2026 (last updated on May 1, 2026), using the core query: (“congenital tufting enteropathy” OR “intestinal epithelial dysplasia”) AND “*EPCAM*”, supplemented with relevant synonyms and spelling variations. Additionally, reference lists of eligible studies were manually reviewed to identify further cases.

### 2.2 Clinical data collection and ethical oversight

For the novel index patient reported in this study, comprehensive clinical data, including demographic characteristics, clinical manifestations, histopathological and electron microscopy findings, and targeted therapeutic interventions, were extracted from electronic medical records. Genomic DNA was extracted from EDTA-anticoagulated peripheral blood. Molecular diagnosis was established via whole-exome sequencing (WES) utilizing the xGen Exome Research Panel v2.0 (Integrated DNA Technologies). The identified variant was subsequently validated by Sanger sequencing in the proband and both unaffected parents to confirm segregation. Written informed consent for the clinical procedures, genetic testing, and subsequent publication of this case report was obtained from the patient's legal guardians. The study protocol concerning the index patient was approved by the Institutional Ethics Committee of Children's Hospital of Nanjing Medical University (Approval No. 202507010-1).

### 2.3 Eligibility and de-duplication

We included molecularly confirmed cases of CTE associated with *EPCAM* variants that provided extractable patient-level data on genotype, nutritional strategy, intestinal transplantation, clinical outcomes (alive or deceased), and major complications (chronic diarrhea, growth failure, malnutrition, electrolyte imbalance, liver damage, infection). Nutritional strategies were specifically categorized as TPN, peripheral parenteral nutrition (PPN), Refused PN, or unknown. Potential overlaps were resolved by cross-checking author lists, institutions, time windows, and patient descriptors. For duplicates, the most informative record was retained. Screening proceeded in two stages: title and abstract review, followed by full-text assessment.

### 2.4 Data extraction and variable definitions

Demographic data, age at onset, family history and zygosity, allele-level variant class, case-level genotype grouping, nutrition status, intestinal transplantation, and vital status were abstracted. When multiple timepoints were available, a single nutrition category was assigned to each patient based on the highest sustained intensity during follow-up, ranked in descending order as TPN, PPN, Refused PN, or unknown.

In this study, all genotypes were grouped into truncating variants (nonsense, large or chromosomal deletions, or compound genotypes with ≥1 truncating allele and without any missense allele) and non-truncating variants (missense, in-frame insertions/deletions, or compound genotypes containing any missense allele). Notably, splicing defects were grouped into non-truncating variants. Given that splicing variants may result in heterogeneous outcomes (e.g., exon skipping or intron retention), and in the absence of transcriptomic data to confirm reading frame disruption for every historical case, we conservatively classified them as non-truncating. This approach avoids overestimating the effect size of the truncating group, although it may dilute the statistical significance. The detailed classification criteria are provided in the supplementary material (Supplement S1).

Nutrition strategy was dichotomized as TPN vs. non-TPN for patient-level analyses. For the genotype–TPN analysis, PPN and refused PN were classified as non-TPN. Cases with unknown nutrition status were classified as non-TPN in the primary analysis and were reclassified as TPN in the sensitivity analysis to test the robustness of the genotype–nutrition association. For the TPN–mortality analysis, cases with unknown nutrition status were excluded because nutrition status served as the exposure variable. Outcomes were coded as alive or deceased. Missing outcome data were addressed using two complementary assumptions: in the primary analysis, unknown outcomes were coded as alive, whereas in the sensitivity analysis, unknown outcomes were coded as deceased.

Major clinical manifestations and complications (chronic diarrhea, growth failure, malnutrition, electrolyte imbalance, liver damage, infection) were abstracted when available. For descriptive summaries, the final pooled cohort was used as the denominator. Unreported or unknown entries were recorded as missing rather than assumed absent, and descriptive proportions were flagged as potentially conservative because of under-reporting. In addition, subgroup comparisons were conducted by geographic region and by World Bank income strata, aiming to describe distributional patterns and generate hypotheses.

To ensure epidemiological rigor and address potential classification bias, geographic origins were re-classified according to the official World Health Organization (WHO) regional groupings. Cases were assigned to the Eastern Mediterranean Region (EMRO), European Region (EURO, including Turkey), Western Pacific Region (WPRO), Region of the Americas (AMRO), South-East Asia Region (SEARO), or African Region (AFRO). This standardized framework was used for all regional comparative analyses and genotype distribution assessments.

### 2.5 Bias mitigation and consistency

Two reviewers independently screened reports and extracted data using standardized coding rules. Disagreements were resolved by consensus. Overlapping patients were handled as described above. Ambiguous cases were coded as unknown.

### 2.6 Statistical analysis

The primary outcome was the association between genotype and the need for TPN. Secondary outcomes were the associations between nutrition status and mortality and between genotype and mortality. Odds ratios (ORs) with 95% confidence intervals (CIs) were calculated from 2 × 2 tables, and statistical significance was assessed using two-sided Fisher's exact tests at *α* = 0.05. Confidence intervals were estimated using the Wald method on the log odds ratio. In tables with zero cells, the Haldane-Anscombe correction (adding 0.5 to each cell) was applied. Effect sizes are reported with 95% confidence intervals, *p* values, and sample sizes for each comparison. Full contingency tables are provided in the Supplement. Analyses were performed in Python 3.11 using pandas and SciPy.

## 3 Results

### 3.1 Case description

**Figure 1:**
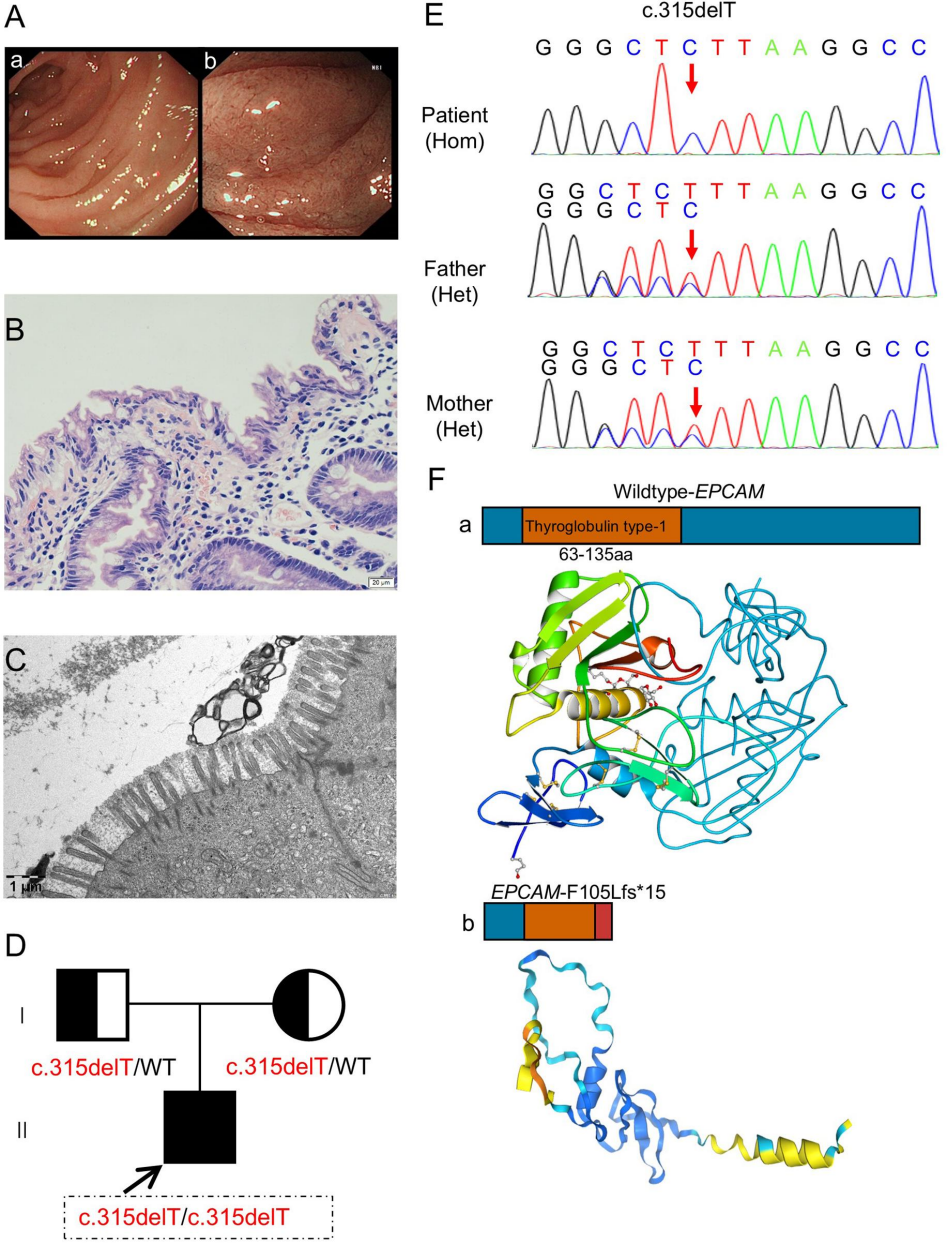
An infant diagnosed with congenital tufting enteropathy carrying a novel pathogenic EPCAM variant. **(A)** Upper endoscopic images showing shortened and blunted villi in the descending duodenum. **(B)** Hematoxylin and eosin staining showing villous atrophy and widened inter-villous spaces. **(C)** Electron microscopy showing shortened, sparse, and flattened microvilli. **(D)** Pedigree showing autosomal recessive inheritance of the EPCAM c.315delT variant. **(E)** Sanger sequencing traces confirming the homozygous c.315delT variant in the patient and heterozygous carrier status in both parents. **(F)** Schematic and structural comparison of wildtype EPCAM and the predicted EPCAM-F105Lfs*15 mutant protein.

The patient, a full-term male infant with a normal birth weight (3.5 kg), exhibited a biphasic clinical deterioration. At 1 month of age, the initial manifestation was poor weight gain without overt watery diarrhea. By 3 months of age, profound growth faltering (weight 3.5 kg, z-score −5.0) prompted the first hospital admission, during which empiric nutritional support failed to yield meaningful weight gain. Classic non-bloody diarrhea only commenced at 5 months of age, accompanied by further weight loss (3.3 kg, z-score −5.5). This severe deterioration prompted readmission, during which mild-to-moderate dehydration and hypoalbuminemia were noted, and symptoms responded poorly to empirical therapy. Upper endoscopy demonstrated shortened and blunted villi in the descending duodenum under white light (Figure 1A). Histology (H&E) showed villous atrophy with widened inter-villous spaces, and electron microscopy revealed markedly shortened, sparse, and flattened microvilli, altogether supporting the diagnosis of congenital tufting enteropathy (Figures 1B,C).

Trio-based whole-exome sequencing with Sanger confirmation and segregation established a homozygous *EPCAM* c.315delT (p.Phe105Leufs*15) in the proband, which, when interpreted under the current ACMG/AMP framework with evidence consistent with PVS1, PM2_Supporting, and PM3_Supporting, was classified as pathogenic (Figures 1D–F). This is a novel pathogenic variant. Despite repeated clinical assessments strongly indicating the need for TPN, the family declined the intervention. The patient ultimately died of severe malnutrition and related complications at 6 months of age. The combination of non-classical onset, an early truncating genotype, and fatal outcome in the absence of TPN provided the clinical rationale for conducting a systematic review and pooled analysis focused on genotype–nutrition–outcome relationships.

### 3.2 Literature search and cohort characteristics

**Figure 2:**
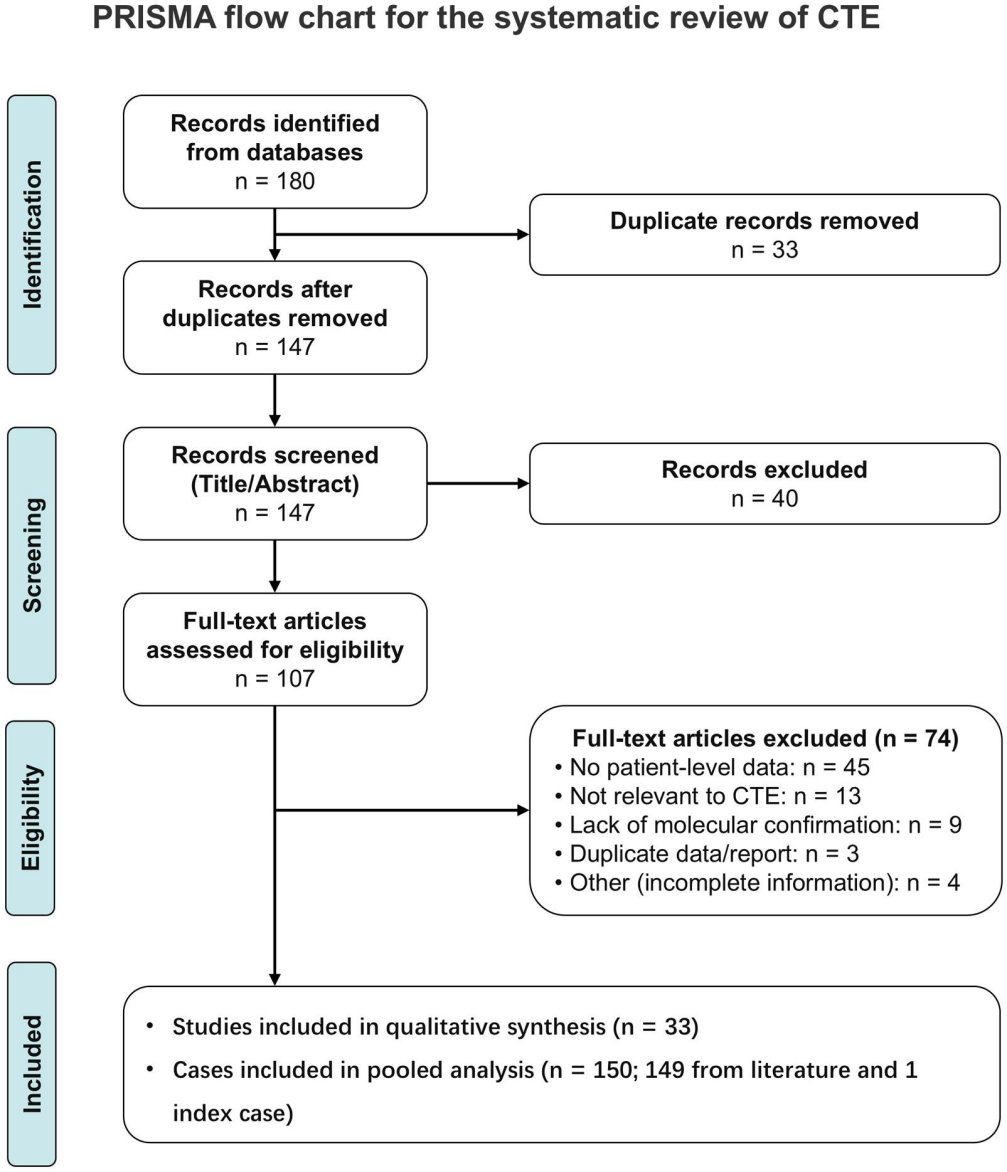
PRISMA flow diagram showing the selection process for the systematic review of congenital tufting enteropathy.

**Figure 3:**
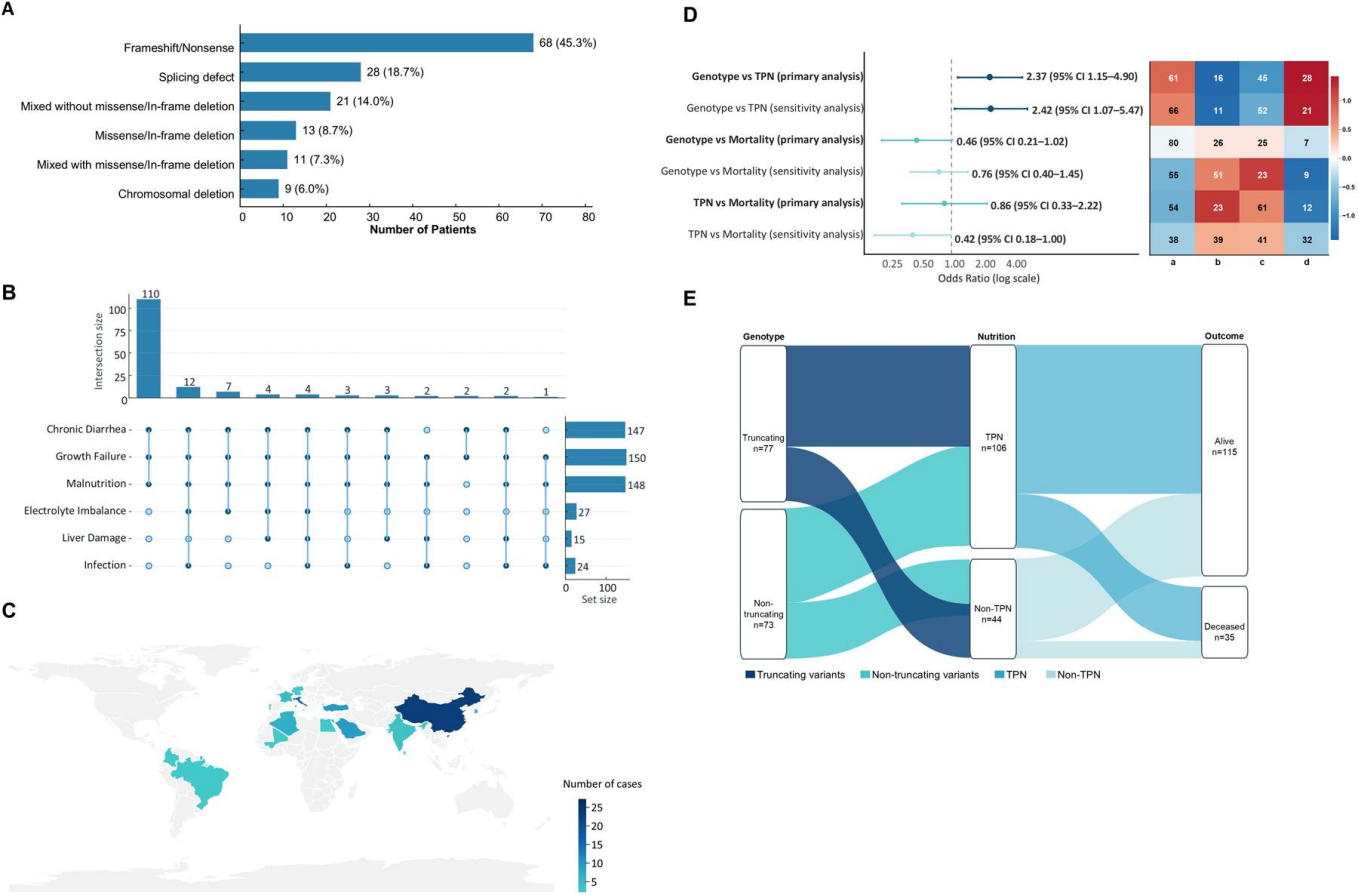
(A–C) Genotypic and clinical characteristics of EPCAM-related congenital tufting enteropathy based on literature review. (D,E). Genotypic and clinical characteristics of EPCAM-related congenital tufting enteropathy based on literature review.

To place the severe phenotype of the index patient in the context of previously reported *EPCAM*-related CTE, we conducted a systematic review and pooled patient-level analysis. The detailed literature screening and selection pathway is illustrated in the PRISMA diagram (Figure 2), yielding a final pooled cohort of 150 molecularly confirmed cases. All included cases are tabulated individually (Supplementary Table S1) (1, 3, 4, 10–38). The results that follow, based on this dataset, first summarize *EPCAM* genotype categories to provide context for subsequent analyses (Figure 3A), and then examine regional distributions, zygosity, country income strata, and the primary and secondary comparisons from the pooled patient-level analysis.

### 3.3 Clinical manifestations and complications

Among the 150 pooled cases, nearly all patients presented with chronic diarrhea (147/150, 98.0%), universal growth failure (150/150, 100%), and malnutrition (148/150, 98.7%). In addition, subsets of patients developed electrolyte imbalance (27/150, 18.0%), liver damage (15/150, 10.0%), and infections (24/150, 16.0%). Taken together, *EPCAM*-related congenital tufting enteropathy is characterized by intractable diarrhea, nutritional insufficiency, and growth retardation as core phenotypes, while metabolic derangements, hepatopathy, and infections represent serious complications that contribute to the disease burden (Figure 3B).

Among the 150 patients with *EPCAM*-related CTE, 35 died, corresponding to an overall mortality rate of 23.3%. The mortality was significantly higher (OR = 2.64, *p* = 0.032) in patients with hepatic or infectious complications (12/31, 38.7%) than in those without such complications (23/119, 19.3%).

### 3.4 Regional profiles and zygosity

Geographic origin was reported across 34 specific countries or territories (Figure 3C). To ensure epidemiological rigor, geographic origins were classified according to the official WHO regional groupings: Eastern Mediterranean Region (EMRO, *n* = 65), European Region (EURO, including Turkey, *n* = 28), Western Pacific Region (WPRO, *n* = 22), Region of the Americas (AMRO, *n* = 10), South-East Asia Region (SEARO, *n* = 10), and African Region/Unspecified (AFRO/Other, *n* = 15). Compared with all other regions combined, the EMRO showed significantly higher homozygosity (90.8% vs. 57.6%; OR 7.22, 95% CI 2.81–18.56; *p* < 0.001) and markedly enriched truncating variants (89.2% vs. 47.1%; OR 9.32, 95% CI 3.82–22.75; *p* < 0.001). The frequency of TPN use (75.4% vs. 67.1%; OR 1.50, 95% CI 0.73–3.10; *p* = 0.284) and mortality among cases with known outcomes (40.0% vs. 24.6%; OR 2.04, 95% CI 0.91–4.58; *p* = 0.099) did not differ significantly between the EMRO and other regions.

### 3.5 Economic strata

Using the World Bank income classification, patient origins were consolidated into high-income countries (HIC, *n* = 76) and non-HIC (*n* = 74; including upper-/lower-middle, low, and unspecified). Compared with cases from non-HICs, those from HICs showed a significantly higher frequency of TPN use (80.3% vs. 60.8%; OR 2.62, 95% CI 1.23–5.65; Fisher's exact *p* = 0.012), while mortality did not differ significantly among those with known outcomes (OR 1.68, 95% CI 0.73–3.88; *p* = 0.228). HIC cases also had a markedly higher proportion of truncating variants (OR 6.19, 95% CI 2.96–12.93; *p* < 0.001) and a greater prevalence of homozygosity (OR 2.34, 95% CI 1.08–5.06; *p* = 0.029). These findings mirror the regional analysis and suggest that regional/economic differences in genotype composition and zygosity may jointly influence nutrition decisions and outcomes.

### 3.6 Genotype-phenotype pooled analysis

In the pooled analysis, truncating genotypes were significantly associated with greater dependence on TPN (61/77, 79.2% vs. 45/73, 61.6%; OR 2.37, 95% CI 1.15–4.90, *p* = 0.020) (Table 1). In the sensitivity analysis, which classified unknown nutrition status as TPN, the result remained consistent (OR 2.42, 95% CI 1.07–5.47, *p* = 0.045). By contrast, among cases with known nutrition status, TPN use was not significantly associated with mortality. In the primary analysis, in which unknown outcomes were coded as alive, the OR was 0.86 (95% CI 0.33–2.22, *p* = 0.82). Under the sensitivity specification coding unknown outcomes as deceased, the association remained statistically non-significant (OR 0.42, 95% CI 0.18–1.00, *p* = 0.066). Genotype was likewise not significantly associated with mortality. In the primary analysis, the genotype–mortality comparison did not reach statistical significance (OR 0.46, 95% CI 0.21–1.02, *p* = 0.056), and the result remained non-significant in the sensitivity analysis (OR 0.76, 95% CI 0.39–1.47, *p* = 0.42) (Figure 3D). The overall flow of patients from genotype group to nutrition category and then to outcome is shown in the Sankey diagram (Figure 3E).

**Table 1:** Summary of genotype classifications and associated phenotypic outcomes (*n* = 150).

Genotype Classification	Cases (*n*)	TPN Dependence, *n* (%)	Mortality, *n* (%)	Major Complications, *n* (%)
Truncating variants	77	61 (79.2%)	23 (29.9%)	Liver damage: 9 (11.7%)
				Infection: 11 (14.3%)
				Electrolyte Imbalance: 13 (16.9%)
Non-truncating variants	73	45 (61.6%)	12 (16.4%)	Liver damage: 6 (8.2%)
				Infection: 13 (17.8%)
				Electrolyte Imbalance: 14 (19.2%)
Total	150	106 (70.7%)	35 (23.3%)	—

## 4 Discussion

Since the initial clinical description of tufting enteropathy in 1994, CTE has been recognized as a rare and severe infantile gastrointestinal disorder characterized by intractable diarrhea and failure to thrive (39). The histopathological hallmarks include villous atrophy, disorganized surface epithelium, and the tufting epithelium (40, 41). A pivotal advancement occurred in 2008 when the *EPCAM* gene, located on chromosome 2p21, was identified as the causative gene (1). It encodes a transmembrane glycoprotein essential for maintaining intercellular adhesion and epithelial barrier integrity. Diverse *EPCAM* variants have been reported, including nonsense, frameshift, and splice-site variants, which typically lead to a loss of protein function (1, 19, 27).

In this study, we identified a novel pathogenic variant, c.315delT (p.Phe105Leufs*15). Mechanistically, this frameshift variant creates a premature termination codon (PTC) in exon 3. According to the canonical 50–55 bp rule, this PTC is strongly predicted to activate the nonsense-mediated mRNA decay pathway, hypothetically leading to functional nullizygosity. Such severe EPCAM deficiency is expected to impair tight-junction integrity through destabilization of claudin-7 and disruption of the E-cadherin/*β*-catenin axis (42, 43). Furthermore, this deficiency compromises intestinal stem cell homeostasis, aggravating the disease phenotype (44). In our case, the markedly shortened, sparse, and flattened microvilli were consistent with severe epithelial barrier disruption and expanded the known mutational spectrum of *EPCAM*.

The rapid clinical deterioration of the index patient, culminating in death in the absence of TPN, raised the possibility that early truncating *EPCAM* variants may be associated with greater intestinal-failure severity. To examine this possibility beyond a single case, we synthesized patient-level data from 150 reported molecularly confirmed cases. The pooled literature suggests a higher concentration of reported CTE cases in regions with high consanguinity rates, such as the EMRO, where founder variants like c.499dupC have been widely reported (1). Our pooled analysis corroborates this geographical predisposition, demonstrating a significant enrichment of truncating variants in the EMRO compared to other regions (OR 9.32, *p* < 0.001). The strong association between truncating variants and homozygous status in this region further supports the founder effect hypothesis (11).

Clinically, CTE typically manifests with intractable diarrhea, malnutrition, and growth failure, which were nearly universal findings in our cohort (2). Some patients develop severe complications, including electrolyte imbalances (18%), liver damage (10%), and infections (16%), all contributing significantly to the overall disease burden. Lifelong TPN remains the cornerstone of management for CTE, compensating for the severe intestinal malabsorption (16, 24, 38). Our pooled analysis suggests that truncating variants are associated with a higher likelihood of TPN dependence (OR 2.37, *p* = 0.020). This finding supports a genotype-informed approach to clinical monitoring and nutritional planning, in which patients carrying truncating variants may warrant earlier evaluation by specialized intestinal-failure and nutrition-support teams.

The overall mortality rate in our cohort was 23.3%, consistent with the severe prognosis associated with CTE, where death is frequently related to complications of malnutrition and liver failure (2). However, mortality rates vary across different studies, including some reports of lower mortality, potentially attributable to regional disparities in healthcare resources (7). This notion is indirectly supported by our finding of a significantly higher TPN utilization rate in high-income countries (80.3%), likely reflecting better healthcare infrastructure and diagnostic capabilities in these settings (19).

In this study, no statistically significant association was found between genotype and mortality. However, the index patient, who carried a truncating variant but did not receive TPN, experienced rapid clinical deterioration and died, which also highlights the importance of TPN in patients with CTE. Therefore, clinical focus should shift from non-modifiable genetic factors to optimizing modifiable medical factors, such as the quality and accessibility of TPN and comprehensive care, which may be crucial for survival.

An apparent clinical paradox exists in our cohort: truncating variants were associated with greater TPN dependence, but genotype was not significantly associated with mortality. This reflects the dual-pathway etiology of CTE complications. In severe truncating phenotypes, profound epithelial barrier dysfunction may lead to severe malnutrition, dehydration, and infection (45). Intensive TPN effectively rescues these patients from early death. However, this obligatory intervention inherently introduces chronic iatrogenic risks. The most notable complications include parenteral nutrition-associated liver disease and central line-associated bloodstream infections (46). Conversely, the subset managed without TPN predominantly features milder, non-truncating variants (28/44, 63.6%). These specific genotypes likely preserve residual EPCAM function and a partially functional microvillous absorptive area. Management for this milder spectrum relies on maximizing residual enteral absorption. Aligned with established clinical consensus for congenital diarrheal disorders, these patients require highly specialized elemental diets. Extensively hydrolyzed or amino acid-based formulas are strictly utilized to minimize luminal osmotic load (47). Even when successfully managed off TPN, patients frequently require intermittent PPN during acute diarrheal exacerbations or rapid somatic growth. This clinical reality underscores a strict therapeutic continuum. Management ranges from life-long TPN in severe phenotypes to tailored enteral rehabilitation for patients with genetically determined residual barrier function.

We acknowledge several critical limitations in our study. First, the pooled analysis is fundamentally limited by its reliance on retrospective, historically and geographically heterogeneous literature data, which carries inherent publication and treatment biases. Therefore, the statistical correlation between truncating variants and TPN dependency must be interpreted as a preliminary clinical observation rather than a definitive prognostic rule derived from a controlled prospective cohort. Second, as mentioned, the absence of functional assays restricts us to predictive mechanistic hypotheses. Finally, the sample size remains limited for robustly evaluating rare secondary outcomes such as mortality. Future multi-center prospective registries and *in vitro* validations are essential.

In conclusion, CTE remains a severe enteropathy with a high overall mortality rate. Alongside identifying a novel pathogenic *EPCAM* variant (c.315delT), our pooled analysis suggests an association between truncating variants and long-term TPN dependence. These findings support genotype-informed nutritional planning. For infants molecularly diagnosed with biallelic truncating variants, clinicians may consider early referral to specialized intestinal-failure and nutrition-support teams, with timely planning for durable venous access and home-TPN programs when clinically indicated. Conversely, for patients with non-truncating variants, a carefully monitored trial of specialized amino acid-based enteral nutrition, supported by temporary PPN when necessary, may help reduce prolonged exposure to total parenteral nutrition. Timely genetic testing may therefore provide an important basis for risk-stratified management in CTE.

## Data Availability

The raw data supporting the conclusions of this article will be made available by the authors, without undue reservation.

## References

[1] Sivagnanam M Mueller JL Lee H Chen Z Nelson SF Turner D Identification of EpCAM as the gene for congenital tufting enteropathy. Gastroenterology. (2008) 135(2):429–37. 10.1053/j.gastro.2008.05.036 18572020 PMC2574708

[2] Goulet O Salomon J Ruemmele F De Serres NP-M Brousse N. Intestinal epithelial dysplasia (tufting enteropathy). Orphanet J Rare Dis. (2007) 2(1):20. 10.1186/1750-1172-2-20 17448233 PMC1878471

[3] Ayyıldız Civan H Leitner C Östreicher I Schneider A-M Cremer M Mayr JA Three novel EPCAM variants causing tufting enteropathy in three families. Children. (2021) 8(6):503. 10.3390/children8060503 34198699 PMC8232273

[4] Oktay MA Çamurdan MO Gürkan ÖE Tehçi BA Döğer E Bideci A. Growth hormone treatment in congenital tufting enteropathy: a case report and literature review. Front Endocrinol. (2025) 15:1492297. 10.3389/fendo.2024.1492297 PMC11821479 39950165

[5] Sivagnanam M Janecke AR Müller T Heinz-Erian P Taylor S Bird LM. Case of syndromic tufting enteropathy harbors SPINT2 mutation seen in congenital sodium diarrhea. Clin Dysmorphol. (2010) 19(1):48. 10.1097/MCD.0b013e328331de38 20009592 PMC6709868

[6] Kellermayer R. Congenital tufting enteropathy in the era of molecular genetics. J Pediatr Gastroenterol Nutr. (2011) 53(3):355. 10.1097/MPG.0b013e318228841a 21691225

[7] Ashworth I Wilson A Aquilina S Parascandalo R Mercieca V Gerada J Reversal of intestinal failure in children with tufting enteropathy supported with parenteral nutrition at home. J Pediatr Gastroenterol Nutr. (2018) 66(6):967–71. 10.1097/MPG.0000000000001894 29334565

[8] Ozler O Brunner-Véber A Fatih P Müller T Janecke AR Arikan C. Long-term follow-up of tufting enteropathy caused by EPCAM mutation p.Asp253Asn and absent EPCAM expression. JPGN Rep. (2021) 2(1):e029. 10.1097/PG9.0000000000000029 37206930 PMC10191536

[9] Nader EA Lambe C Talbotec C Pigneur B Lacaille F Garnier-Lengliné H Outcome of home parenteral nutrition in 251 children over a 14-y period: report of a single center. Am J Clin Nutr. (2016) 103(5):1327–36. 10.3945/ajcn.115.121756 27030532

[10] Fang Y Luo Y Yu J Chen J. A case of severe malnutrition infant with neonatal onset intractable diarrhea. BMC Pediatr. (2020) 20(1):133. 10.1186/s12887-020-1999-0 32293360 PMC7087373

[11] Salomon J Espinosa-Parrilla Y Goulet O Al-Qabandi W Guigue P Canioni D A founder effect at the EPCAM locus in congenital tufting enteropathy in the Arabic Gulf. Eur J Med Genet. (2011) 54(3):319–22. 10.1016/j.ejmg.2011.01.009 21315192

[12] Zhao R Yang F Chen X Fu H Li G Cheng L A novel compound-heterozygous EPCAM mutation in congenital tufting enteropathy. Arch Med Sci. (2022) 18(6):1700–4. 10.5114/aoms/155185 36457962 PMC9710291

[13] Shakhnovich V Dinwiddie D Hildreth A Attard T Kingsmore S. A novel compound-heterozygous epithelial cell adhesion molecule mutation in tufting enteropathy. J Pediatr Gastroenterol Nutr. (2017) 64(1):e14–6. 10.1097/MPG.0000000000000629 25383784 PMC4426084

[14] Namuslu ŞN Sevinç E Ekmen S Akan K Metin H. A novel homozygote EpCAM gene mutation in Turkish neonate with tufting enteropathy. Exp Appl Med Sci. (2024) 5(4):201–6. 10.46871/eams.1522547

[15] Thoeni C Amir A Guo C Zhang S Avitzur Y Heng YM A novel nonsense mutation in the EpCAM gene in a patient with congenital tufting enteropathy. J Pediatr Gastroenterol Nutr. (2014) 58(1):18–21. 10.1097/MPG.0000000000000106 24048167

[16] Fang Y Luo Y Xu L Yu J Chen J. Case series of eight congenital tufting enteropathy patients and literature review. Clin Genet. (2025) 108(1):69–74. 10.1111/cge.14702 39980129

[17] Mantoo MR Malik R Das P Yadav R Nakra T Chouhan P. Congenital diarrhea and enteropathies in infants: approach to diagnosis. Indian J Pediatr. (2021) 88(11):1135–8. 10.1007/s12098-021-03844-z 34292522

[18] Surti A Paredes SL Gremse D Parsell K. Congenital tufting enteropathy a rare cause of failure to thrive in an infant—a case report. Am J Med Sci. (2023) 365:S220–1. 10.1016/S0002-9629(23)00421-4

[19] Pathak SJ Mueller JL Okamoto K Das B Hertecant J Greenhalgh L EPCAM Mutation update: variants associated with congenital tufting enteropathy and lynch syndrome. Hum Mutat. (2019) 40(2):142–61. 10.1002/humu.23688 30461124 PMC6328345

[20] Sivagnanam M Schaible T Szigeti R Byrd RH Finegold MJ Ranganathan S Further evidence for EpCAM as the gene for congenital tufting enteropathy. Am J Med Genet Part A. (2010) 152A(1):222–4. 10.1002/ajmg.a.33186 20034091 PMC6691968

[21] d’Apolito M Pisanelli D Faletra F Giardino I Gigante M Pettoello-Mantovani M Genetic analysis of Italian patients with congenital tufting enteropathy. World J Pediatr. (2016) 12(2):219–24. 10.1007/s12519-015-0070-y 26684320

[22] Salomon J Goulet O Canioni D Brousse N Lemale J Tounian P Genetic characterization of congenital tufting enteropathy: epcam associated phenotype and involvement of SPINT2 in the syndromic form. Hum Genet. (2014) 133(3):299–310. 10.1007/s00439-013-1380-6 24142340

[23] Bodian DL Vilboux T Hourigan SK Jenevein CL Mani H Kent KC Genomic analysis of an infant with intractable diarrhea and dilated cardiomyopathy. Mol Case Stud. (2017) 3(6):a002055. 10.1101/mcs.a002055 PMC5701300 28701297

[24] Güvenoğlu M Şimşek-Kiper PÖ Koşukcu C Taskiran EZ Saltık-Temizel İN Gucer S Homozygous missense epithelial cell adhesion molecule variant in a patient with congenital tufting enteropathy and literature review. Pediatr Gastroenterol Hepatol Nutr. (2022) 25(6):441. 10.5223/pghn.2022.25.6.441 36451688 PMC9679307

[25] Tan QK-G Cardona DM Rehder CW McDonald MT. Identification of EPCAM mutation: clinical use of microarray. Clin Case Rep. (2017) 5(6):980–5. 10.1002/ccr3.914 28588851 PMC5457984

[26] Yan W Xiao Y Zhang Y Tao Y Cao Y Liu K Monogenic variants in four cases of neonatal-onset watery diarrhea and a mutation review in east Asia. Orphanet J Rare Dis. (2021) 16(1):383. 10.1186/s13023-021-01995-y 34503561 PMC8427875

[27] Zhou Y-Q Wu G-S Kong Y-M Zhang X-Y Wang C-L. New mutation in EPCAM for congenital tufting enteropathy: a case report. World J Clin Cases. (2020) 8(20):4975–80. 10.12998/wjcc.v8.i20.4975 33195669 PMC7642537

[28] Esposito MV Comegna M Cernera G Gelzo M Paparo L Berni Canani R NGS Gene panel analysis revealed novel variants in patients with rare congenital diarrheal disorders. Diagnostics. (2021) 11(2):262. 10.3390/diagnostics11020262 33567694 PMC7915612

[29] Tang W Huang T Xu Z Huang Y. Novel variants in EPCAM cause congenital tufting enteropathy. J Clin Gastroenterol. (2018) 52(1):e1–6. 10.1097/MCG.0000000000000739 27875355

[30] Hassan K Sher G Hamid E Hazima KA Abdelrahman H Al Mudahka F Outcome associated with EPCAM founder mutation c.499dup in Qatar. Eur J Med Genet. (2020) 63(10):104023. 10.1016/j.ejmg.2020.104023 32735948

[31] Gonzalez-Hakspiel LC Wilches-Cuadros MA Nausa-Suárez PA Fernández F Patiño-Ascencio P Manrique-Guerrero A Severe congenital diarrhea secondary to tufting enteropathy. Case report. Case Rep. (2022) 8(1):41–50. 10.15446/cr.v8n1.90883

[32] Wang S-N Fu Y-J Lu X-L Miao S-J Zhang P Wang L Three patients with new variants in the EPCAM variant gene for congenital tufting enteropathy and a mutation review in China: a case report. Transl Pediatr. (2024) 13(8):1486–95. 10.21037/tp-24-97 39263299 PMC11384436

[33] Al-Mayouf SM Alswaied N Alkuraya FS AlMehaidib A Faqih M. Tufting enteropathy and chronic arthritis: a newly recognized association with a novel EpCAM gene mutation. J Pediatr Gastroenterol Nutr. (2009) 49(5):642–4. 10.1097/MPG.0b013e3181acaeae 19820410

[34] Pêgas KL Cambruzzi E Ferrelli RS Silva CSD Guedes RR Adami M Tufting enteropathy with EpCAM mutation: case report. J Bras Patol Med Lab. (2014) 50(3):234–7. 10.5935/1676-2444.20140021

[35] Ko JS Seo JK Shim JO Hwang SH Park HS Kang GH. Tufting enteropathy with EpCAM variants in two siblings. Gut Liver. (2010) 4(3):407–10. 10.5009/gnl.2010.4.3.407 20981223 PMC2956358

[36] Zhang L An H Zhang J Fu H Zhang L Zhang J. A case of congenital tufting enteropathy caused by EPCAM gene mutation. Chin J Clin Exp Pathol. (2022) 38(9):1143–5. 10.13315/j.cnki.cjcep.2022.09.029

[37] Yang M Xie Y Zhang H. A case of congenital tufting enteropathy with EpCAM gene complex heterozygous mutation (c.491 + 1G > A; c.352_353ins CACC). J Sichuan Univ(med Sci). (2022) 53(3):493–6. 10.12182/20220560109 PMC10409424 35642160

[38] Yuan C Huang Z Liu Y Wu J. Congenital tufting enteropathy caused by mutation of EPCAM gene:a case report and review of literature. J Clin Pediatr. (2018) 36(8):618–20. 10.3969/j.issn.1000-3606.2018.08.013

[39] Reifen RM Cutz E Griffiths AM Ngan BY Sherman PM. Tufting enteropathy: a newly recognized clinicopathological entity associated with refractory diarrhea in infants. J Pediatr Gastroenterol Nutr. (1994) 18(3):379–85. 10.1002/j.1536-4801.1994.tb11192.x 8057225

[40] Das B Okamoto K Rabalais J Kozan PA Marchelletta RR McGeough MD Enteroids expressing a disease-associated mutant of EpCAM are a model for congenital tufting enteropathy. Am J Physiol-gastrointest Liver Physiol. (2019) 317(5):G580–91. 10.1152/ajpgi.00098.2019 31433211 PMC6879886

[41] Cai C Chen Y Chen X Ji F. Tufting enteropathy: a review of clinical and histological presentation, etiology, management, and outcome. Gastroenterol Res Pract. (2020) 2020:1–10. 10.1155/2020/5608069 PMC7530495 33029133

[42] Keller L Werner S Pantel K. Biology and clinical relevance of EpCAM. Cell Stress. (2019) 3(6):165–80. 10.15698/cst2019.06.188 31225512 PMC6558934

[43] Ladwein M Pape U Schmidt D Schnolzer M Fiedler S Langbein L The cell-cell adhesion molecule EpCAM interacts directly with the tight junction protein claudin-7. Exp Cell Res. (2005) 309(2):345–57. 10.1016/j.yexcr.2005.06.013 16054130

[44] Das B Okamoto K Rabalais J Young JA Barrett KE Sivagnanam M. Aberrant epithelial differentiation contributes to pathogenesis in a murine model of congenital tufting enteropathy. Cell Mol Gastroenterol Hepatol. (2021) 12(4):1353–71. 10.1016/j.jcmgh.2021.06.015 34198013 PMC8479479

[45] Lacaille F Gupte G Colomb V D’Antiga L Hartman C Hojsak I Intestinal failure-associated liver disease: a position paper of the ESPGHAN working group of intestinal failure and intestinal transplantation. J Pediatr Gastroenterol Nutr. (2015) 60(2):272–83. 10.1097/MPG.0000000000000586 25272324

[46] Hartman C Shamir R Simchowitz V Lohner S Cai W Decsi T ESPGHAN/ESPEN/ESPR/CSPEN guidelines on pediatric parenteral nutrition: complications. Clin Nutr. (2018) 37(6):2418–29. 10.1016/j.clnu.2018.06.956 30033173

[47] Thiagarajah JR Kamin DS Acra S Goldsmith JD Roland JT Lencer WI Advances in evaluation of chronic diarrhea in infants. Gastroenterology. 2018;154(8):2045–2059.e6. 10.1053/j.gastro.2018.03.067 29654747 PMC6044208

