# Supplementary material for: Genotype-phenotype correlations in EPCAM-associated congenital tufting enteropathy: a case report and systematic review
Source: Front Pediatr. 2026 May 21;14:1830464. doi: 10.3389/fped.2026.1830464 (PMC13233504; doi:10.3389/fped.2026.1830464)
Supplement: Supplementary file 2 [file Supplementaryfile1.docx]

# Supplementary 1. Genotype Grouping and Two-Bin Consolidation

1. Data sources and original six-category grouping

At the patient level, allele annotations (HGVS, ProteinChange) and per-allele functional labels MutationClassification(Col 1/Col 2) (e.g., *Nonsense, Frameshift, Deletion, Splicing defect, In-frame deletion, Start-loss, Stop-loss*) were harmonized into the following six Genotype Group categories for consistent coding:

1. Frameshift/Nonsense: frameshift and/or nonsense only;
2. Chromosomal deletion: exon-level/large segment/chromosomal deletions (typically expected to disrupt the reading frame or remove essential domains);
3. Splicing defect: canonical ±1/±2 splice-site variants or well-supported non-canonical splice-disrupting variants;
4. Missense/In-frame deletion: missense only, or in-frame indel only;
5. Mixed without missense/In-frame deletion: mixed combinations without missense (e.g., splicing plus in-frame exon deletion);
6. Mixed with missense/In-frame deletion: mixed combinations that include missense, or in-frame variants co-occurring with other types.

2. Two-group analysis framework and consolidation rules: truncating variants vs non-truncating variants

For patient-level comparisons, the six categories were consolidated into two analysis bins as defined below.

**Definitions (patient level)**

**Truncating variants:** genotypes containing frameshift, nonsense, or exon-level/large/chromosomal deletions expected to cause a frameshift or remove essential domains.

**Non-truncating variants:** genotypes containing missense, in-frame indels/exon deletions, splicing variants, or mixed genotypes classified as non-truncating under the predefined conservative rules.

**Mapping from six categories to two bins**

Frameshift/Nonsense → **Truncating variants**

Chromosomal deletion → **Truncating variants** (deletions expected to cause a frameshift or essential-domain loss)

Splicing defect → **Non-truncating variants**

Missense/In-frame deletion → **Non-truncating variants**

Mixed without missense/In-frame deletion → **Non-truncating variants**

Mixed with missense/In-frame deletion → **Non-truncating variants**

**Rationale**

This consolidation is effect-driven. Frameshift, nonsense, and deletions predicted to cause frameshifts or remove essential domains are aligned with loss-of-function mechanisms and therefore define the truncating-variant group. Missense, in-frame, and splicing variants—and their mixtures—exhibit broader functional heterogeneity and are grouped as non-truncating variants to preserve analytic consistency and interpretability across a heterogeneous case literature.

# Supplementary 2. Operational definitions of nutrition categories and handling of Unknown nutrition status

Scope and grouping

At the patient level, nutrition was normalized to four categories: TPN, PPN, Refused PN, and Unknown. For the genotype–TPN analysis, PPN and Refused PN were classified as non-TPN. Cases with Unknown nutrition status were classified as non-TPN in the primary analysis and reclassified as TPN in the sensitivity analysis. For the TPN–mortality analysis, cases with Unknown nutrition status were excluded because nutrition status was the exposure variable.

Operational definitions

1. TPN (total parenteral nutrition): Parenteral nutrition delivered via central venous access or explicitly described as long-term/central-line TPN.
2. PPN (peripheral parenteral nutrition): Parenteral nutrition delivered via peripheral access with no evidence of central-line TPN.
3. Refused PN: PN was recommended but declined by caregivers/patient; no sustained PN was subsequently delivered.
4. Unknown: Nutrition status not explicitly reported or insufficient for adjudication to any of the above subcategories.

Adjudication rules

Assignments were made from source text verbatim: documented TPN → TPN; otherwise, documented PPN → PPN; otherwise, Refused PN if PN was advised but declined; otherwise, Unknown.

Composition of the non-TPN group in the primary genotype–TPN analysis

| **Category**  **(within Non-TPN)** | **n** | **Non-TPN(%)** | **Notes** |
| --- | --- | --- | --- |
| PPN | 24 | 54.5% | Peripheral PN without evidence of central-line TPN |
| Refused PN | 8 | 18.2% | PN recommended but declined; no subsequent sustained PN |
| Unknown | 12 | 27.3% | Not explicitly reported or insufficient to adjudicate |
| Total (Non-TPN) | 44 | 100% | — |
